# Introducing the Next Era in Assessment

**DOI:** 10.5334/pme.1551

**Published:** 2025-01-09

**Authors:** Alina Smirnova, Michael A. Barone, Sondra Zabar, Adina Kalet

**Affiliations:** 1 Clinical Assistant Professor, Department of Family Medicine, University of Calgary, Canada; 2 President and CEO of the American Board of Pediatrics, US; 3 A Professor of Medicine and Director of the Division of General Internal Medicine and Clinical Innovation at the NYU Grossman School of Medicine, New York, New York, USA; 4 A Professor at the Medical College of Wisconsin, Wisconsin, USA

## Abstract

In this introduction, the guest editors of the “Next Era in Assessment” special collection frame the invited papers by envisioning a next era in assessment of medical education, based on ideas developed during a summit that convened professional and educational leaders and scholars.

The authors posit that the next era of assessment will focus unambiguously on serving patients and the health of society, reflect its sociocultural context, and support learners’ longitudinal growth and development. As such, assessment will be characterized as transformational, development-oriented and socially accountable. The authors introduce the papers in this special collection, which represent elements of a roadmap towards the next era in assessment by exploring several foundational considerations that will make the next era successful. These include the equally important issues of (1) focusing on accountability, trust and power in assessment, (2) addressing implementation and contextualization of assessment systems, (3) optimizing the use of technology in assessment, (4) establishing infrastructure for data sharing and data storage, (5) developing a vocabulary around emerging sources of assessment data, and (6) reconceptualizing validity around patient care and learner equity. Attending to these priority areas will help leaders create authentic assessment systems that are responsive to learners’ and society’s needs, while reaping the full promise of competency-based medical education (CBME) as well as emerging data science and artificial intelligence technologies.

Schuwirth and van der Vleuten (2020) posited that there have been at least three clearly identifiable eras in medical education assessment over the past sixty years, each representing shifts in the dominant discourses [1]. Starting in the 1960s, assessment was regarded mainly as an issue of measurement for the purpose of distinguishing among individuals and therefore was focused on highly standardized testing with strong psychometric qualities. In the 1990s, the focus on workplace assessment led to embracing human judgment in assessment and the role of assessment for learning. Most recently, beginning in the 2000’s thought leaders have vigorously promoted a longitudinal systems view manifested as programmatic assessment embracing the multifaceted and complex nature of assessment. Conceptions of validity in assessment have similarly evolved from a focus on establishing test characteristics toward making a chain of evidentiary arguments, including most recently a focus on social accountability and equity [2,3].

These shifts are not discrete categorical transitions, but represent an evolution in prioritization of the multiple elements of assessment. Most recently, the COVID-19 pandemic has deeply influenced the way we view assessment, highlighting many challenges to the field, bringing issues of diversity, equity and inclusion (EDI) to center stage, as well as accelerating implementation of new technologies such as remote performance based assessment and artificial intelligence [4,5,6,7,8,9]. A new era of assessment is therefore rapidly emerging.

Health professions education functions as a complex adaptive system. When seeking changes, we must carefully consider the many foundational forces acting on this system. Given the growing consensus that time variable competency based medical education is the framework best fit to meet the evolving needs of the profession and society, we must consider better integration of assessment across the continuum of medical education and embrace the affordances of data sciences and artificial intelligence. In addition to these forces, we must also consider the seismic shifts in healthcare organization and financing, biomedical and technical breakthroughs, and the evolving experiences, expectations, and desires of future healthcare professionals [10,11]. Addressing these complex assessment challenges has the potential to guide rapid and effective adaptation to the changing healthcare and education landscape.

In this introduction to the special collection, entitled “Next Era in Assessment”, we explore the fundamental forces currently reshaping assessment in medical education and envision a pathway to the future which explores and explicates these forces. We introduce this special collection of papers borne from an invitational summit, which brought together medical education scholars and organizational leaders in May 2022 with the goal to tackle the most pressing challenges in assessment (Technical Report, Appendix). Together, the papers in the “Next Era in Assessment” reflect the collective urgency to accelerate the evolution of assessment in medical education toward an increasingly authentic patient- and learner-centered system that prepares future health professionals to care for patients and communities with excellence and agility for the length of their careers in medicine [11].

We begin by exploring some of the broad foundational forces shaping the next era of assessment in medical education. These forces vary in intensity, from gentle breezes to gale-force winds. Some originate within health professions and assessment disciplines, while others come from outside, shaping and reshaping the context in which assessment takes place. By analyzing patterns in these forces, we can better understand and guide their influence on assessment in this next era. The papers in this special collection identify and discuss these forces, outline a vision for the next assessment era, and suggest a roadmap forward.

## The need to address trust and power in assessment

It is difficult, but critical, to maximize trust in an assessment system. Systems of assessment become trustworthy when they is co-produced, transparent, accountable to the public, and viewed by learners as authentic (i.e. true to the professional activities expected of physicians) [12]. They must also be facilitated by assessors who are eager facilitative educators and are recognized as role models [13,14]. Currently, lack of trust is a significant barrier to optimizing assessment [13,15]. In some cases, trainees view assessments as an irrelevant hurdle to their training. For example, medical students spend money on test preparation courses and materials and residents take time off their clinical training because they do not trust the curriculum prepares them for high stakes exams. The current assessment system can be associated with a “striving to survive” narrative, where some trainees feel compelled to merely perform or may be driven to game the assessment system. Ideally, an assessment system would scaffold learners and assessors to encourage honest self-assessment and a growth orientation, i.e., a “striving to thrive” narrative [16].

## The need to consider context and implementation of assessment

Assessment continues to be significantly impacted by global events such as pandemics, local weather related events, including hurricanes, floods and wild fires, political polarization, war, and disparities associated with climate change and rapid technological advancements [5,8,17,18,19]. The recent SARS and COVID-19 pandemics highlighted the need for health professionals to be highly adaptable. Experienced health professionals were redeployed to care for patients outside their domain of expertise [20,21,22], or very inexperienced clinical trainees volunteered to serve as front line medical care providers [23]. An assessment system that does not robustly integrate context will not be able to account for how shifting and unpredictable events impact on the competence of future generations of physicians [19].

An effective medical education assessment system must be flexible, dynamic and adapt rapidly to change. Most importantly, it must respond to and enable evolving learning needs, changing expectations for societal accountability and collaboration with an ever-enlarging group of stakeholders [24]. The movement toward *precision medical education* exemplifies the type of flexibility and outcomes orientation that could frame a highly functional system of assessment facilitated by optimal use of emerging information technology [10,25,26]. Precision education is defined as a “systematic approach that integrates longitudinal data and analytics to drive precise educational interventions that address each individual learner’s needs and goals in a continuous, timely, and cyclical fashion, ultimately improving meaningful educational, clinical, or system outcomes” [26]. In such a system, information about learners and practicing professionals is gathered across curricula, learning environments and engagements with specific patient populations, program/institution and national levels of medical education. This information travels with and scaffolds the self-directed education and identity formation of learners longitudinally over their training and practice journeys. As more robust assessment systems develop, educational program leaders and regulators will have the information to make valid and defensible promotion decisions about individuals across multiple authentic contexts and settings. Ideally, this will support the highest quality and safest care for individual patients while addressing the needs of communities and the public’s health. Such a system could enable an unprecedented, robust quality improvement and research agenda to inform health professions education and its role in patient outcomes [27]. Implementing and iteratively improving such a system will require a long-term concerted effort, good stewardship and vigilance regarding unintended positive and negative consequences of this systems change. It will require a high level of trust among learners, educators, the profession and society.

Implementation challenges in assessment are not new [28,29]. Implementation of assessment necessitates broad efforts to longitudinally align objectives, instructional and assessment strategies within the curriculum [1]. Full realization of this approach not only requires a common mental model of the assessment system’s purposes, but also the necessary resourcing and leadership of a challenging change management and implementation science effort [30]. As van der Vleuten stated, *“Educational innovation is an issue of change management rather than having the right ideas”* (Technical report, Appendix). By recognizing the interconnectedness of the education system with many others, including the healthcare, political and social systems, we can begin to delineate how medical education operates within highly complex and contextualized learning environments, only a small piece of which is directly under our control. Without tackling the inevitable challenges that come with implementation, assessment will continue to stagnate.

## The need to build systematic approaches and infrastructure for technological advances

The explosion in the quantity of available data and advances in data science technology leading to the scaling of artificial intelligence also present dramatic opportunities and threats [8,31]. The field of assessment is now challenged to translate new sources of data and new data elements into meaningful assessment and evaluation information on individuals and systems of care. The next era of assessment must focus on improving data infrastructure and data-related policies, including the ethical use of data and its validity at many levels. Old measurement frameworks may not sufficiently incorporate new competencies (e.g. adaptive competence) nor new types of data (e.g. electronic medical record, dashboards) to support robust validity arguments in this next era. Assessment developers in the next era must consider new technologies and sources of data – both their development and use – while limiting unintended negative consequences of technology use, including assessment fatigue, burden on assessors, lack of trust, and waste of resources.

Barriers to data sharing between and within institutions or with researchers pose additional challenges to new assessment strategies [32,33]. Without a systematic approach to storing and sharing data about learners it would be impossible to reap the benefits afforded by new technology and sources of data, such as longitudinal assessment across institutions and stages of training.

## The need to rethink validity as a social imperative

The issues of trust, equity, diversity and inclusion in assessment as well as new technologies and data sources have brought to light existing problems with overreliance on process and psychometric properties independent of the tool/instrument design [2,3]. Moreover, the dominant discourses of validity are partially responsible for sustaining systemic inequities in healthcare and medical education [34]. More recently, however, there has been an emphasis on the role of validity in supporting the social imperative, an approach which emphasizes social accountability, validity integrated into assessment process, and inclusion of qualitative data as a source of validity evidence [35]. To address the systemic inequities and rebuild the trust of learners and society, we must critically examine how current conceptualizations of validity can reflect the values of today’s society and medical education community in the next era of assessment.

## Will the next era of assessment be transformative, development-oriented, and socially accountable?

We predict that the next era of assessment will be defined by [1] an explicit recognition of the interconnectedness among the health professions education system and the surrounding healthcare, political and social systems, and [2] a robust growth-, longitudinal-, and development- oriented program of assessment for learning. In these ways, assessment shifts from simply being a context sensitive adjunct to instruction and towards becoming the foundation of transformative socially accountable learning [36].

Shifting assessment practices toward interconnectedness requires recognizing that medical education operates within complex adaptive educational, clinical and social systems. Ideally, these systems can align around the goals of both supporting flexible and dynamic assessment responsive to individual learners’ needs and meeting public needs by achieving optimal patient outcomes through shared responsibility for longitudinal development of people (learners, faculty, patients) and systems (organizations) [37]. An assessment system that is transformative will influence trainees’ clinical practice by stimulating reflection and learning. It will also impact whole training programs by providing insights into trainees’ performance in practice, and shape medical education systems by aligning learning with patient care.

A transformative assessment system must center around a development orientation that promotes a growth mindset at all stages of training and practice and the education system (individual, program/institution, and national). Shifting towards a development orientation requires highly collaborative and cooperative handoffs between each training stage – from undergraduate to graduate medical education and from graduate medical education to practice. In a development-oriented assessment system, various types of assessments should be used to identify areas for improvement for individuals as they progress through training and their careers, as well as for training programs and institutions as they adapt to change.

At its core, the assessment system should be accountable to both patients and learners at every level of medical education. A socially accountable assessment system embeds the core social values of relevance, quality, effectiveness, and equity at all levels of assessment [38]. On the program/institutional level, socially equitable assessment systems align training and assessment with population needs, developing a competent and diverse workforce that addresses current specialty and geographic shortages [39,40]. They also ensure all graduates can effectively create an equitable, diverse, and inclusive workplace [41]. On the (inter)national level, a socially accountable assessment system should be transparent and deliberately support the mission of health professions education and healthcare by fostering safe learning environments and allocating resources to ensure equitable training opportunities [42]. It should, ultimately provide value to the public and broadly address EDI issues [40]. Social accountability in the next era will be reflected not only in the validity frameworks underpinning assessment but also in the harmonization of core assessment elements across international borders, enhancing international cooperation and reducing barriers for training, emergency response, and workforce needs [43].

## A roadmap towards the next era in assessment

The papers in this special collection represent elements of a roadmap towards a transformative, development-oriented and socially accountable era of assessment by exploring several foundational considerations to make the next era of assessment successful. These include (1) accountability, trust and power in assessment, (2) implementation and contextualization of assessment, (3) harnessing the use of technology in assessment, (4) improving infrastructure for data sharing and open source data, (5) a new vocabulary for assessment data, and (6) validity in the next era of assessment.

1. In exploring accountability, trust and power in assessment, Caretta-Weyer et al. [44] focus on building trust through increasing transparency about the collection and use of learner assessment data and breaking down many of the negative power relationships in the learning environment to promote a growth-oriented mindset. The current emphasis on test performance creates a competitive, transactional, achievement -oriented mindset amongst learners, which contrasts with the reflective, lifelong learners that physicians are expected to become.
2. In their paper on implementation and contextualization of assessment, Kassam et al. [45] address theoretical perspectives on how contextual patterns and complexity can be considered when implementing the next era of assessment. They emphasize that assessment systems work best when co-produced with stakeholders, including patients, healthcare professionals, patients, electronic health record vendors (designing metrics), health system leaders (integrating assessment into workflows), educators (ensuring assessors have skills and attitudes consistent with authentic assessment), and accreditors (incentivizing best practices systems implementation).
3. Krumm et al. [47] provide key questions to ask when seeking to harness technology for assessment. They discuss useful frameworks that can help strategically integrate technology into assessment practice.
4. Sebok-Syer et al. [46] present a roadmap and a five-step process for building infrastructure to leverage assessment data, including clinical data, to support individual and educational improvement. Data governance, user engagement and funding are included in the model as continuous processes. Their scalable roadmap can be implemented from local to national levels, helping individuals, organizations and institutions limit their engagement in resource consuming low-yield efforts.
5. With the new sources of data being used for assessment, Krumm et al. aim to unlock their potential by proposing an organizing vocabulary for categorizing types of data [47]. This allows for more purposeful use of data by the broader medical education assessment community.
6. Validity evidence underpins trust in assessment data and the decisions based on that data. It is important that our conceptions of validity reflect the values of today’s medical education community. In their paper, Kinnear et al. [48] propose ways of making validity arguments in support of assessment that are socially accountable, inclusive, fair and equitable.

This special collection aims to advance conversations in the medical education assessment community while placing patients and learners at the center of our concerns. We share this discussion and collection of papers to embrace new possibilities, challenge assumptions, expand assessment horizons, and affirm the gains of previous eras of assessment. Transformative, development-oriented, and socially accountable assessment will strengthen both the education system and patient care. We hope these papers will engage trainees, educators, and assessors in fruitful discussions, self-reflection, and co-production to usher assessment into the next era.

## Additional File

The additional file for this article can be found as follows:

10.5334/pme.1551.s1 Appendix. Next Era in Assessment.
